# Uncovering the genetic basis of gluten aggregation parameters by genome-wide association analysis in wheat (*Triticum aestivum* L.) using GlutoPeak

**DOI:** 10.1186/s12870-022-03874-5

**Published:** 2022-10-21

**Authors:** Zhengqing Wu, Hongxia Qiu, Zhaoran Tian, Congcong Liu, Maomao Qin, Wenxu Li, Pan Yang, Yao Wen, Baoming Tian, Fang Wei, Zhengfu Zhou, Zhensheng Lei, Jinna Hou

**Affiliations:** 1 Henan Institute of Crop Molecular Breeding, Henan Academy of Agricultural Sciences, Postgraduate T & R Base of Zhengzhou University, Zhengzhou, 450002 China; 2 School of Agricultural Sciences, Zhengzhou University, Zhengzhou, 450001 China; 3 National Key Laboratory of Wheat and Maize Crop Science, Henan Agricultural University, Zhengzhou, 450002 China; 4 Shennong Laboratory, Zhengzhou, 450002 Henan China

**Keywords:** Wheat, Gluten aggregation properties, GlutoPeak parameters, GWAS

## Abstract

**Background:**

Numerous studies have shown that gluten aggregation properties directly affect the processing quality of wheat, however, the genetic basis of gluten aggregation properties were rarely reported.

**Results:**

To explore the genetic basis of gluten aggregation properties in wheat, an association population consisted with 207 wheat genotypes were constructed for evaluating nine parameters of aggregation properties on GlutoPeak across three-year planting seasons. A total of 940 significant SNPs were detected for 9 GlutoPeak parameters through genome-wide association analysis (GWAS). Finally, these SNPs were integrated to 68 non-redundant QTL distributed on 20 chromosomes and 54 QTL was assigned as pleiotropic loci which accounting for multiple parameters of gluten aggregation property. Furthermore, the peak SNPs representing 54 QTL domonstrated additive effect on all the traits. There was a significant positive correlation between the number of favorable alleles and the phenotypic values of each parameter. Peak SNPs of two novel QTL, *q3AL.2* and *q4DL*, which contributing to both *PMT* (peak maximum time) and A3 (area from the first minimum to torque 15 s before the maximum torque) parameters, were selected for KASP (Kompetitive Allele Specific PCR) markers development and the KASP markers can be used for effectively evaluating the quality of gluten aggregation properties in the association population.

**Conclusion:**

The rapid and efficient GlutoPeak method for gluten measurement can be used for early selection of wheat breeding. This study revealed the genetic loci related to GlutoPeak parameters in association population, which would be helpful to develop wheat elite lines with improved gluten aggregation through molecular marker-assisted breeding.

**Supplementary Information:**

The online version contains supplementary material available at 10.1186/s12870-022-03874-5.

## Introduction

As one of the three major staple crops, wheat provides more than 20% of the calories and proteins for the global population [1]. Currently, high quality, as the same as high yield, has become the major objective for wheat improvement. Further, with the increasing population worldwide and the critical requirement of healthy diet for improving the chronic disease caused by modern poor eating habits. The quality divergence among wheat genotypes, to a great extent, is determined by the content and composition of wheat seed storage proteins (SSP) [2]. Wheat SSP is mainly consisted of gliadin and glutenin in endosperm which largely determine the rheological properties of dough [3]. Among them, gliadin (40%-50%) is a group of monomer proteins while glutenin (30%-40%) is consist of polymer polypeptide chains interacted with disulfide bonds. These two classes of SSP form the gluten network which endows dough with viscoelasticity in wheat [4–7]. Gliadin mainly affects the extensibility of the dough, and glutenin mainly affects dough elasticity [8, 9]. The strength of interactions among gliadin and glutenin molecules determine the aggregation characteristic of the gluten. Principal component analysis of durum wheat with different glutenin to gliadin ratios confirmed that gluten aggregation index differentiated high quality genotypes from those of low quality. Consequently gluten aggregation properties can be used as an important indicator to evaluate wheat grain quality [10].

The gluten aggregation properties evaluated by GlutoPeak (BRABENDER TECHNOLOGIE GMBH & CO. KG, DUISBURG, GERMANY) consisted with nine parameter values: *PMT* (peak maximum time); *BEM* (maximum torque); *AM* (torque 15 s before the *BEM*); *PM* (torque 15 s after the *BEM*); A1 (area from the start of the test to the first maximum); A2 (area from the first maximum to the first minimum); A3 (area from the first minimum to *AM*); A4 (area from *AM* to *BEM*); and A5 (area from *BEM* to *PM*). It has been proved that the nine parameters of gluten aggregation properties tested by GlutoPeak are significantly correlated with those tested by traditional instruments, such as farinograph and extensometer [11, 12]. The parameter value of *PM* is highly correlated with gluten strength [13]. *PMT*, as one of the quality parameters for aggregation properties, highly correlated with the mixograph peak time (*r* = 0.90) and gluten index (*r* = 0.88) in durum, and it can be adopted for differentiating varieties with different gluten strength effectively. *PMT* negatively correlated with dough viscosity [14]. Studies on the association of gluten aggregation properties with the content of gliadin, SDS-soluble protein, glutenin, and glutenin macropolymer revealed that the value of *PMT* was correlated with the content of glutenin and glutenin polymer, therefore, decrease the viscosity of dough. Another GlutoPeak parameter, *BEM*, is significantly correlated with the contents of gliadin and SDS-soluble protein [15]. There was a significant positive correlation between the value of *BEM* and water absorption measured by Farinograph in Canadian wheat accessions (*r*^*2*^ = 0.97), the GlutoPeak can be used to estimate water absorption of dough [16]. The GlutoPeak parameter *AM* presented the high correlation with loaf volume (*r* = 0.77). The *AM* value can be adopted for prediction of bread volume to a certain extent, that is useful to screen high quality wheat for bread-making [17]. The parameter *PM* which measured by GlutoPeak with whole-meal wheat flour was highly correlated with gluten strength [13]. Therefore, GlutoPeak is an effective tool for rapid evaluating the wheat quality traits and selecting the elite lines at the early breeding stage.

The aggregation property of gluten directly determines its strength, and furtherly, affects the processing quality of dough. To date, a large number of studies have reported the influence of environmental and developmental factors, such as sprouting duration, storage temperature of mature grains and the period of storage time after harvest, on gluten aggregation properties [18, 19]. Sprouting led to significant increase of *PMT* and decrease of *BEM* and aggregation energy [18, 19]. However, few researches revealed the genetic basis of wheat gluten aggregation properties. Studies indicated that the broad-sense heritability (*H*^*2*^) of GlutoPeak parameters, such as *PMT*, *AM* and A3, was higher than 70%, which indicated that the gluten aggregation properties were mainly determined by genetic factors [20]. Therefore, elucidating the genetic basis and developing molecular markers of the major genetic loci for gluten aggregation properties are beneficial to wheat quality improvement. In the present study, an association population with 207 wheat genotypes was constructed, and the panel of genotypes was planted under three environments. Nine parameters related with gluten aggregation properties were measured by GlutoPeak, the phenotypic variance of all parameters ranged from 7.55% to 24.21%. Genome-wide association study (GWAS) was conducted for dissecting the QTL (Quantitative Trait Locus) related with 9 parameters related with gluten aggregation properties from GlutoPeak. Finally, a total of 68 QTL, integrated from 940 single nucleotide polymorphisms (SNPs), were identified. Two QTL, *q3AL.2* and *q4DL*, were selected for developing the kompetitive allele specific PCR (KASP) markers referencing the genomic sequence of the SNPs significantly associated with these two QTL. These two KASP markers significantly influenced the phenotype values of *PMT* and A3 (*P* < 0.05). The PLATZ transcription factor gene, *TraesCS3A02G497600*, which reported its important role in seed development and carbohydrate synthesis in crops, was recognized as the candidate gene for one of the novel QTL affecting wheat seed development and tissue differentiation on 3AL.

## Results

### Phenotypic variation of the association population

**Fig. 1:**
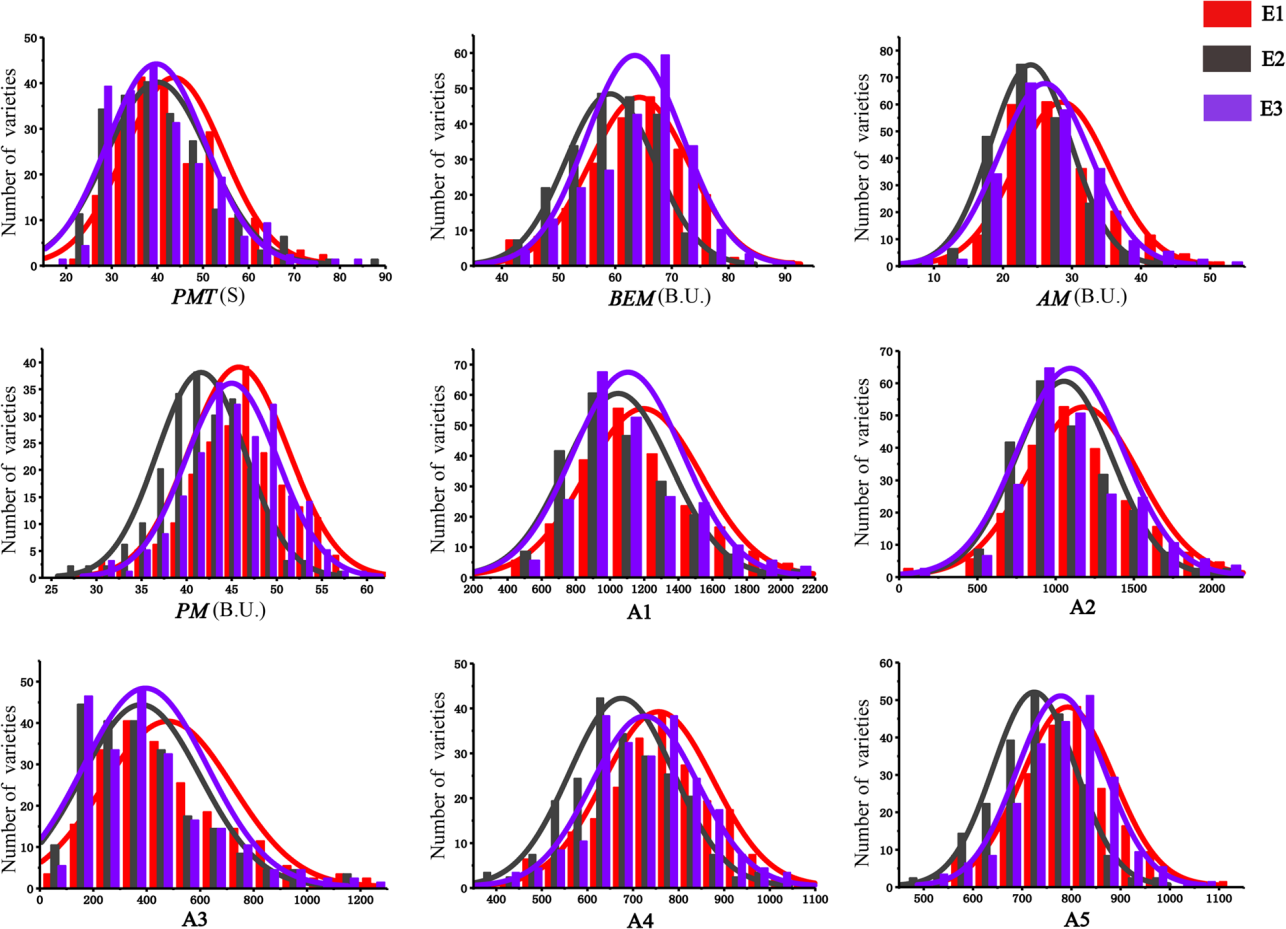
Distribution of nine parameters evaluated by GlutoPeak in the association population under three environments. E1: Yuanyang in 2017–2018, E2: Yuanyang in 2018–2019, E3: Yuanyang in 2019–2020. Different environments are distinguished by different colors. The values of each trait were presented in X-axis. *PMT*: peak maximum time; *BEM*: maximum torque; *AM*: torque 15 s before *BEM*; *PM*: torque 15 s after *BEM*; A1: the area from the start of the test to the first maximum; A2: the area from the first maximum to the first minimum; A3: the area from the first minimum to *AM*; A4: the area from *AM* to *BEM*; A5: the area from *BEM* to *PM*

Nine parameters (*PMT*, *BEM*, *AM*, *PM*, A1, A2, A3, A4 and A5) represented the gluten aggregation properties were evaluated by GlutoPeak in the association population. Statistical analysis of the phenotypic values showed that the value of GlutoPeak parameters in association population varied across different environments, the phenotypic variance of all parameters ranged from 7.56% to 24.21%, with the variation coefficients ranged from 12.56% to 62.61% in three environments (Tables S1 and S2). The values of all parameters demonstrated as the normal distribution under three environments (Fig. 1). Correlation analysis showed that the nine parameters were positively correlated with each other (Table S3). Based on ANOVA, it is found that the genotype (G), environment (E) and the genotype by environment interaction (G x E) significantly influenced all the parameters (*P* < 0.001). The broad-sense heritability (*H*^*2*^) of all parameters were ≥ 0.85 (Table 1). The parameter A3 displayed the largest coefficient of variation (CV = 62.61%), while *PMT* was highly positively correlated with A3 (*r* = 0.98). *PMT* and A3 displayed the highest broad-sense heritability (*H*^*2*^ = 0.95) which means the genetic factor was the main contributor to these traits and was conducive to genetic loci identification.

### Genome-wide association study (GWAS) of gluten aggregation properties

**Fig. 2:**
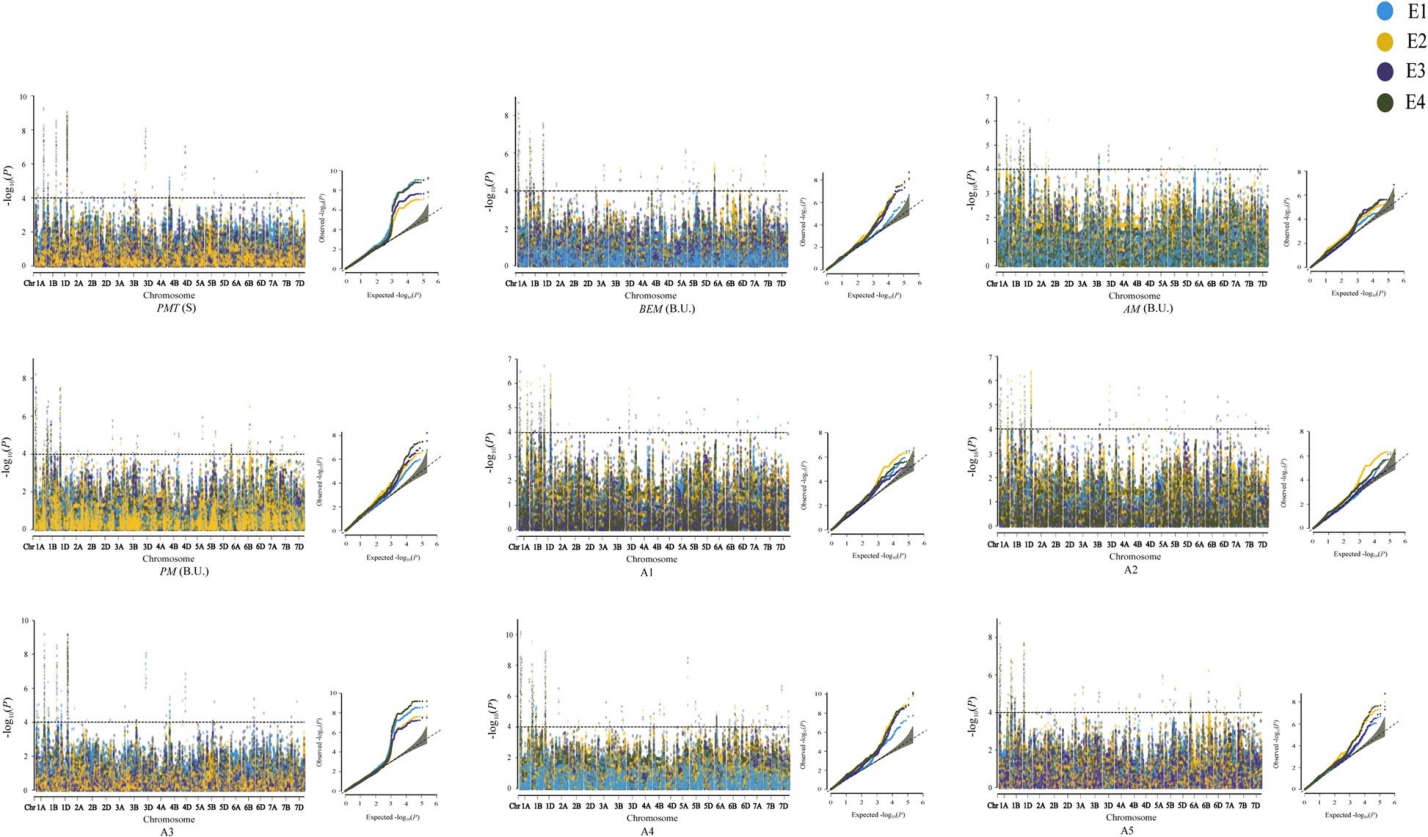
Manhattan plot and Q-Q plot of GlutoPeak parameters. E1, E2 and E3 represent Yuanyang in 2017–2018, 2018–2019 and 2019–2020 planting seasons, respectively, and E4 represents BLUP value of the three environments. Black horizontal dotted line indicates significance threshold line (-log_10_*P* = 4)

Our previous research genotyped 207 genotypes in the association population using Wheat Breeders 660 K Axiom® array, and 244,507 SNP were identified. Population structure analysis indicated that the association population can be classified into two groups [21]. After quality control, the set 224,706 SNPs were used for further analysis in TASSEL v5.0 [22]. Genetic loci associated with all of the nine parameters were analyzed by GWAS (Fig. 2 and Table S4). A total of 68 QTLs were detected significantly associated with nine GlutoPeak parameters (-log_10_ *P* value ≥ 4). For specific parameter of gluten aggregation properties, there were 14, 25, 20, 25, 31, 27, 20, 38 and 29 QTLs for *PMT*, *BEM*, *AM*, *PM*, A1, A2, A3, A4, and A5, respectively. These QTLs distributed on all chromosomes except 5D (Table S5). Sixty-eight significant SNPs with the highest phenotypic variation from each QTL were selected as the Peak SNPs for further analysis.

### Evaluation the additive effect of Superior alleles on gluten aggregation

**Fig. 3:**
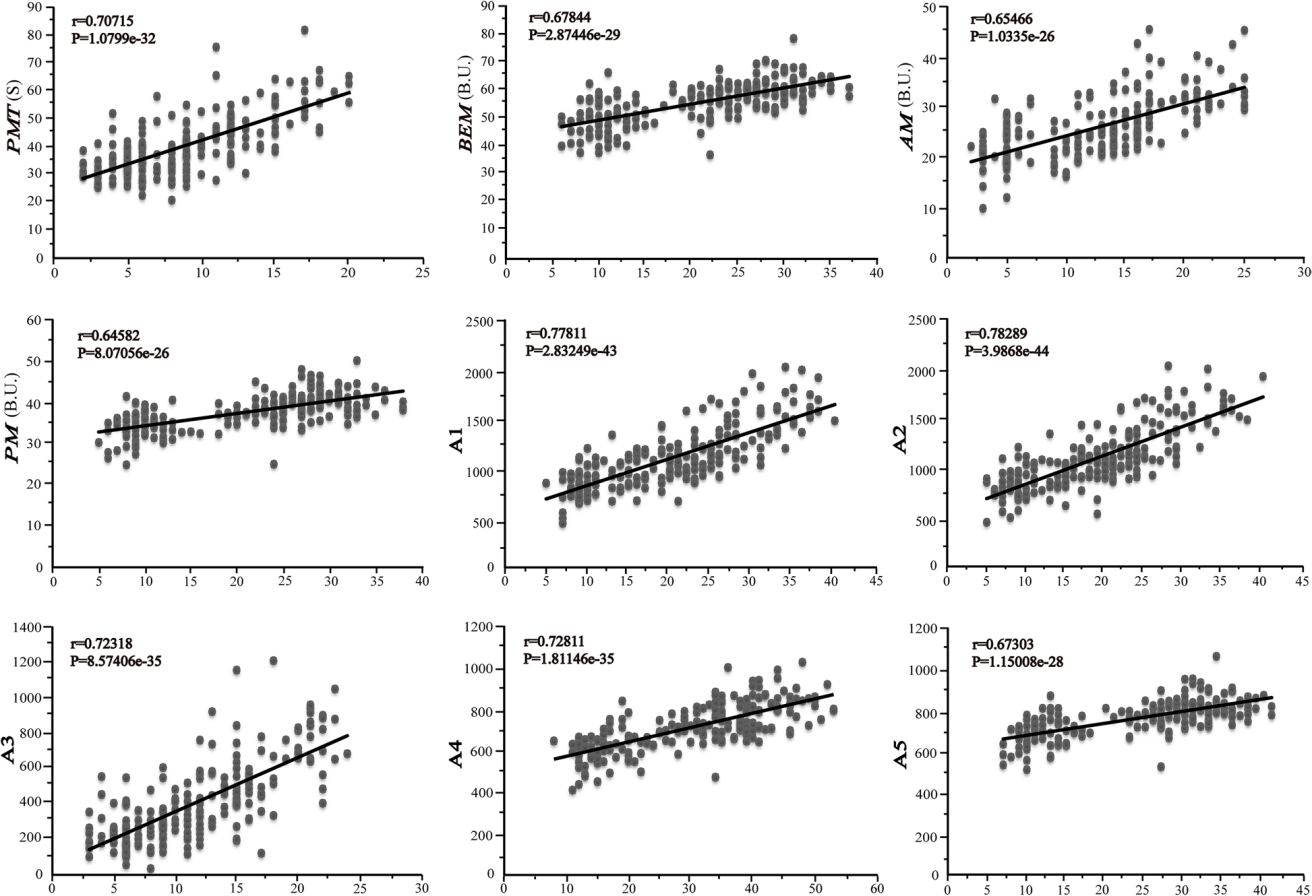
Superior alleles additive effect on nine GlutoPeak parameters in the association population. The Y-axis indicated the phenotypic values of 207 wheat genotypes, and the X- axis represented the number of superior alleles in the genotype, accordingly

The superior alleles were defined as the alleles conferred with higher phenotypic values of gluten aggregation property parameters than their counterparts; vice versa, the inferior alleles associated with lower phenotypic values. As reported in our previous work, the superior alleles were assigned with the score of ‘1’, while the inferior alleles were scored ‘0’. To evaluate the additive effect of the superior alleles on GlutoPeak parameters, 68 peak SNPs were selected for genotyping the genotypes in association population. The correlation between the scores and the average value of each variation was displayed in the scatter plot (Fig. 3). The nine parameters of GlutoPeak showed a significant positive correlation between the number of superior alleles and the phenotypic values in association population (*r* > 0, *p* < 0.001). The genotypes with more superior alleles demonstrated higher phenotypic values of gluten aggregation properties.

### Analysis the pleiotropic SNPs affecting both *PMT* and A3

**Fig. 4:**
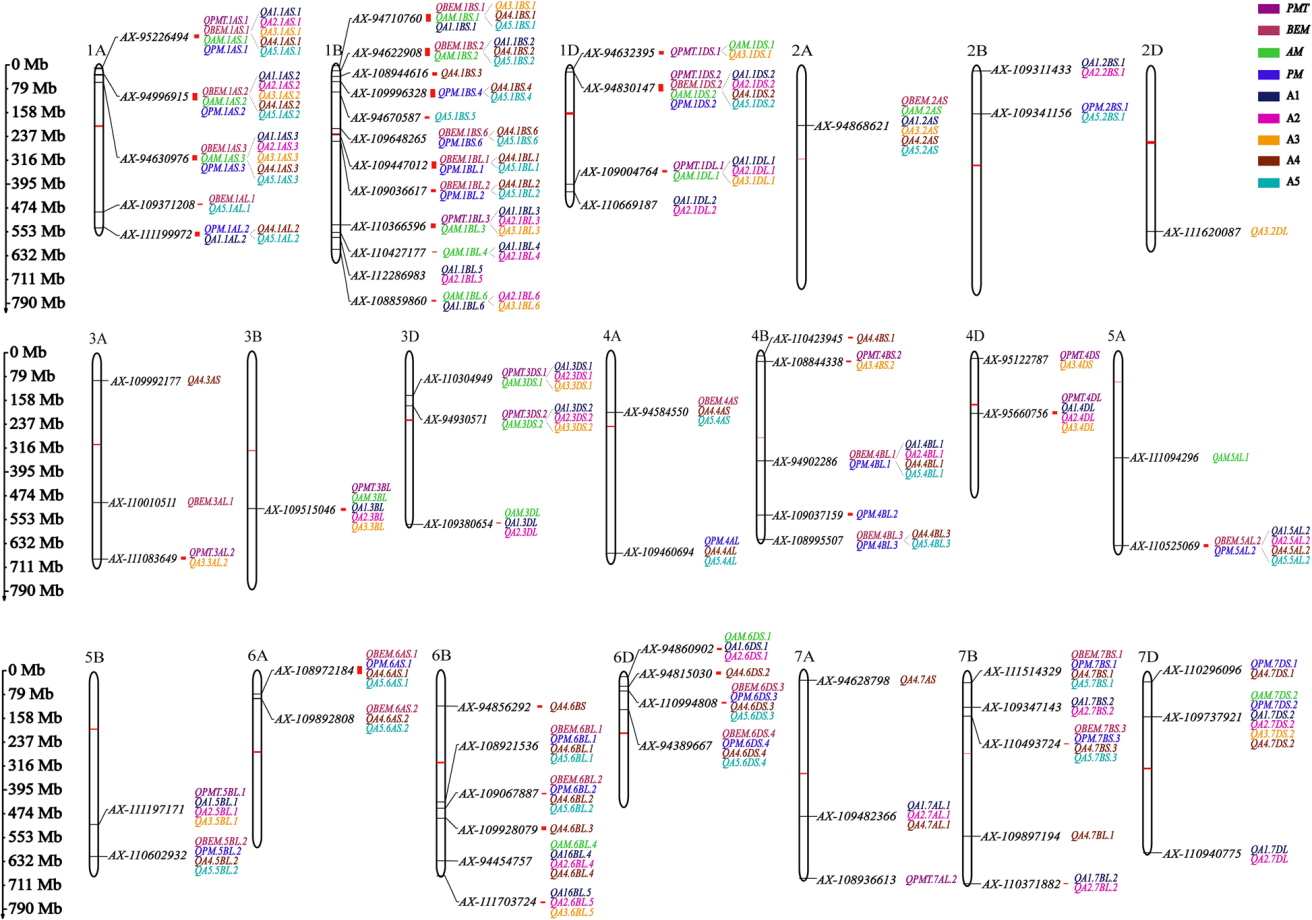
Physical location of all the QTL for gluten aggregation properties measured by GlutoPeak. Red lines on chromosomes represented centromeres, different colors represent QTL for different traits, and red rectangles represent QTL intervals. The physical position was marked with the black lines on the left of the figure. The peak SNP of each QTL were labeled to the right of each chromosome

The 68 QTL were located on the physical map referencing the position of the Peak SNPs. Among them, 54 QTL account for two or more parameters, and these QTL demonstrated the tendency of distributing in clusters on the chromosomes (Fig. 4, Table S6). Accordingly, the peak SNPs from the 54 QTL were assigned as pleiotropic SNPs. Nearly almost of the SNPs (53/54) significantly effect on 9 gluten aggregation property parameters (*t* – test, *P* < 0.001), except for the SNP *AX-109004764* (*q1DL.1*: 408 Mb ~ 416 Mb) on chromosome 1DL (Table S4). The phnotypic difference of all the 9 parameters was analyzed based on the allelic polymorphism of 54 pleiotropic SNPs. Among them, 11 SNPs associated with both of *PMT* and A3. The SNP, A*X-95226494,* which is the peak SNP for QTL on 1AS.1 accounted for all the GlutoPeak parameters, with the phenotypic variation of 7.79%-18.51%. The peak SNPs, *AX-110366596*, *AX-109515046*, *AX-110304949* and *AX-94930571*, of the QTL located on 1BL.3, 3BL, 3DS.1 and 3DS.2, respectively, affected *PMT*, *AM*, A1, A2 and A3 simultaneously, and explained 7.92%—20.56% of the phenotypic variation. Peak SNPs, *AX-111083649*, *AX-108844338* and *AX-95122787*, located on 3AL.2, 4BS.2 and 4DS, respectively, affected *PMT* and A3, explaining 7.82%—12.65% of the phenotypic variation. *AX-95660756* (*q4DL*: 204 Mb-215 Mb) and *AX-111197171* (*q5BL.1*: 531 Mb) affected *PMT*, A1, A2, and A3, explaining 8.84–15.35% of the phenotypic variation. *AX-94632395* (*q1DS.1*: 513,067-11 Mb) was detected contributing to the parameters *PMT*, *AM*, and A3 and explained 8.26%—12.36% of the phenotypic variation.

### Development of KASP markers

**Fig. 5:**
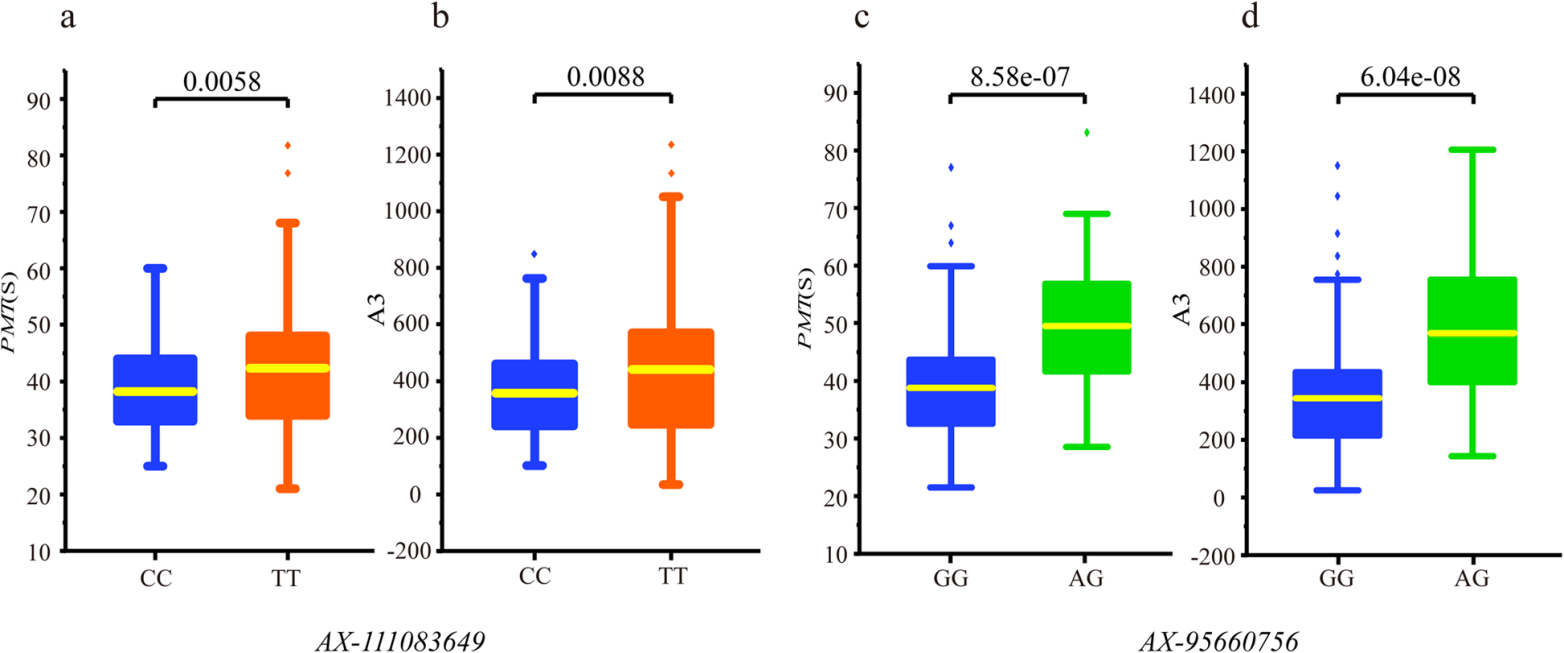
Phenotypic difference of *PMT* and A3 values with different genotype of two KASP markers in association population. Phenotypic variation between the genotype of CC/TT in the SNP AX-111083649 (**a**, **b**) and GG/AG in AX-95660756 (**c**, **d**). The genotype was labeled under each column and the *P*-value was indicated above the columns

Comparing 68 QTL detected in the present study with genetic loci for quality traits revealed by previous research, 38 QTL were found to be newly discovered, which had not been reported in previous studies. Two of them which effected on PMT and A3 simultaneously, located on chromosome 3A and 4D, respectively, were selected to development KASP markers (Table S7). The phenotype of *PMT* and A3 were significantly associated with the genotypes of two KASP markers (*AX-111083649* and *AX-95660756*) in the association population (*t* – test, *P* < 0.01, Fig. 5 and Table S8). The present result indicated that the KASP markers could identify the gluten aggregation properties effectively and be used for selecting wheat lines with suitable *PMT* and *A3* parameters.

### Prediction of the candidate genes

**Fig. 6:**
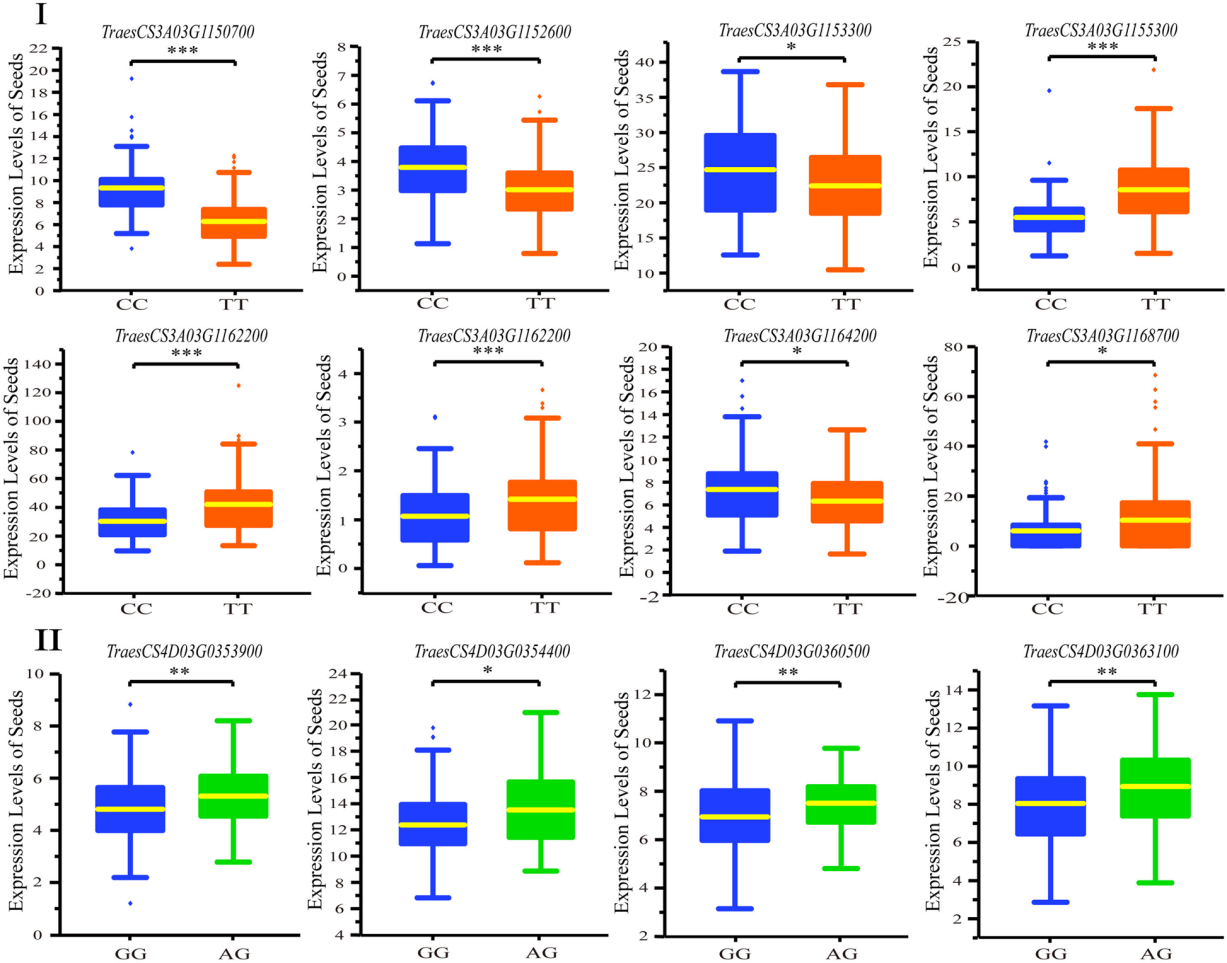
Variation of annotated gene expression between different genotypes in the association population. I: Comparison of expression level of 8 candidate genes in the QTL *q3AL.2* with the peak SNP *AX-111083649*. II: Comparison of expression level of 4 candidate genes in the QTL *q4DL* with the peak SNP *AX-95660756*. The genotypes were displayed in X- axis; the expression level (represented by Fragments Per Kilobase per Million) of each gene were demonstrated in Y- axis. The *P* values were marked on the top of each pair of comparison columns

The expression level of the genes underneath the QTL, *q3AL.2* (Peak SNP: *AX-111083649*) and *q4DL* (Peak SNP: *AX-95660756*) were detected in developing grains. The 10Mbp genomic sequence flanking each of 5Mbp from down- and up- stream of the peak SNPs was used for gene annotation, and 38 genes were identified expression in developing seeds. However, only eight genes (*TraesCS3A03G1150700, TraesCS3A03G1152600, TraesCS3A03G1153300, TraesCS3A03G1155300, TraesCS3A03G1162200, TraesCS3A03G1162900, TraesCS3A03G1164200, and TraesCS3A03G1168700*) *and* and four genes (*TraesCS4D03G0353900, TraesCS4D03G0354400, TraesCS4D03G0360500 and TraesCS4D03G0363100*), from *q3AL.2* and *q4DL*, respectively, were detected significant expression divergence between different genotypes in *AX-111083649* and *AX-95660756* in the association population (Fig. 6).

The function of the eight differentially expressed genes were analyzed. Among all the genes annotated from the two QTL, *TraesCS3A03G1168700,* which is a PLATZ transcription factor and plant-specific zinc-dependent DNA-binding protein specifically expressed in seeds (Table S9). Previous studies have revealed that the PLATZ gene family played an important role in seed development and carbohydrate synthesis in crops [23, 24]. Therefore, it was recognized the most possible candidate gene for *PMT* and *A3* parameter values.

## Discussion

To date, studies have confirmed that gluten aggregation is highly related with rheological properties of dough. The divergence of dough processing quality was largely determined by the content and composition of gluten [25]. Therefore, exploring the genetic basis of gluten aggregation properties facilitates accurately optimizing processing quality in wheat. In the present study, GlutoPeak was adopted for gluten aggregation related parameters evaluation in an association population consisted with 207 wheat genotypes. Statistical analysis of the obtained data demonstrated that all parameters were correlated with each other (*r* range 0.24 to 0.98, *P* < 0.01), and the phenotypic variation was mainly controlled by genetics. Considering the highest broad-sense heritability (*H*^*2*^ = 0.95) of *PMT* and A3, and significant correlation (*r* = 0.98, *P* < 0.01) between the two parameters, the *PMT* and A3 were recognized the most important parameters for evaluating gluten aggregation properties.

A great deal of studies focused on revealing the genetic basis of wheat qualities traits, such as the content of glutenin, gliadin and wet gluten. However, the research on the genetic characterization of gluten aggregation was relatively less. Comparison of the 68 QTL revealed in the present study with the quality related genetic loci reported by previous studies, interestingly, 30 gluten aggregation properties related QTL were located in the same or adjacent regions to previously reported quality traits related QTL in wheat (Table S10). Previous study detected 64 QTL through genetic analysis of 6 quality traits at different sowing dates. Among which, *GENE-0412_338, Excalibur_rep_c109101_115* and *Kukri_c17467_2711* accounting for protein content, water absorption and grain hardness at late sowing time, and these three QTL were co-located with *q1AS.1, q2BS.1* and *q3BL* in this study; five QTL, *IAAV1194, wsnp_JD_c10389_11059599, RFL_Contig148_359, TA003913-0402* and *RAC875_c16731_2004* related with protein content and dough stability time at normal sowing time, and they were co-located with *q1BS.1, q2BS.1, q3DL, q6AS.1* and *q6BL.5* in the present study [26]. Seven multi-trait loci, AX*-94694208*, *AX-94685030*, *AX-94926263*, *AX-95075882*, *AX-94694411*, *AX-95203056* and *AX-94613317* which located on chromosomes 1A, 1D, 3A, 4B, 7B and 7D, respectively, contributing to wheat grain quality traits and dough rheological properties were co-located with *q1AS.2, q1DS.1, q1DS.2, q3AS, q4BL.3, q7BS.1* and *q7DL* detected in our present study [27]. The 2 marker-trait associations (Clone ID: 871,955 and 1,049,708) obtained from GWAS on spring bread wheat were co-localized with 2 QTL (*q2DL* and *q4BS.1*) revealed in this study [28]. A study conducted GWAS using the models of MLM and FarmCPU for wheat quality traits and reported three QTL on 1B, and these three QTL co-located with *q1BS.2, q1BL.5* and *q1BL.6* screened in the present study [29]. A total of 15 QTL on chromosomes 1A, 1B, 4B, 4D, 5A, 6A, 6B, 7A, 7B in the present study were co-localized with the QTL for grain protein content, gluten strength and sedimentation volume revealed by previous genetic analyses [30–33]. The QTL on chromosome 1A, 1B, 3A, 4B, 4D and 6A co-located with QTL for end-use quality in spring wheat [34]. Our previous study reported the genetic loci for gluten aggregation properties in a RIL populations, and mapped QTL on 1D, 3A, 4D which was repeatedly detected in association population in the present study [20]. In the present study, 68 QTL were dissected, among which 38 were speculated to be the novel genetic loci. These results prove that GWAS for GlutoPeak parameters was reliable, furtherly, more genetic loci would be explored for better understand the genetic basis of gluten aggregation properties in wheat.

GlutoPeak parameters can be used as important indexes to evaluate wheat quality [35–37]. In the present association population, analysis showed that five parameters, *PMT*, *AM*, A1, A2 and A3, were highly correlated with each other (*r* ≥ 0.85), while other four parameters, *BEM*, *PM*, A4 and A5 were highly correlated with each other (*r* > 0.85). However, the correlation between the above mentioned two sets of parameters was relative lower. It’s has been reported that the parameters, *PMT* and A3, were significantly associated with gluten strength and other quality traits in wheat [38]. The study on gluten aggregation properties should pay more attention to the parameters of *PMT*, *AM*, A1, A2, and A3. Therefore, two SNPs from the novel detected QTL affecting both *PMT* and A3 were selected for developing KASP markers. These two markers significantly affected the phenotypic values of *PMT* and A3 in the association genotypes, which indicated that the elite alleles in *AX-111083649* and *AX-95660756* could improve the gluten aggregation properties of wheat. Our present study would assist for early selection in wheat breeding and benefit to genetic improvement of gluten aggregation properties in wheat.

## Materials and methods

### Plant materials

The association population consisted with a total of 207 wheat genotypes mainly composed of local varieties, historical cultivars and breeding parents collected from Henan, Shaanxi, Sichuan and other provinces of China, and the genotypes originated from Australia, Mexico, Russia and other countries, was planted in Yuanyang (Henan province, China, YY, E113°97′, N35°05′) across three planting seasons (2017–2018, 2018–2019 and 2019–2020). The panel of 207 cultivars was collected by the Henan Province Crop Germplasm Bank and The International Maize and Wheat Improvement Center (CIMMYT). The authors declare the total permissions to use the collections. Each genotype was sown by hand in a plot with four rows of 2 by 0.2 m, and the surveyor's rods with 10 cm spaces labels were used to ensure the appropriate space between adjacent plants. All the genotypes were cultivated and treated according to the local management. Each of the genotypes was sown in October and harvested in May of the next year.

### Collection of the phenotypic data

The whole-meal flour of 207 wheat genotypes were prepared by LM3100 (Perten, Sweden) and stored in cold room (4℃). The moisture content of flour was tested by IM9500 (Perten, Sweden) based on AACC (American Association of Cereal Chemists) approved method 46–30.01. The gluten aggregation properties of whole-meal flour under three environments were measured by GlutoPeak (BRABENDER TECHNOLOGIE GMBH & CO. KG, DUISBURG, GERMANY). First, required amount of each sample for measurement on GlutoPeak was calculated based on the moisture content, then 0.5 mol/L CaCl_2_ solution was added into the sample as the activator (GlutoPeak software was used to calculate the sample amount and the required solution volume). Finally, the jacketed sample cup was heated by circulating water at 34℃ and the rotation speed of the paddle was set at 1900 rpm to extract wheat gluten [39]. The whole-meal flour in the jacketed sample cup was mixed with the activator, and the gluten was separated by rapid stirring of the paddle. Accordingly, the aggregation of gluten exerted resistance on the paddle, and a peak curve which reflecting the gluten aggregation properties emerged on the equipment software [40]. The gluten aggregation properties of each sample were defined as the average value of two replicates.

## Statistical analysis

The Microsoft Excel 2016 software was used to conduct the descriptive statistical analysis (minimum value, maximum value, average value, standard deviation, etc.) for the phenotypic data of the association population in three environments. The origin 2017 was used to draw the frequency distribution map, and the "Lme4" software package from R software (R × 64 3.6.3) (R Core Team, 2019) was used to calculate the best linear unbiased predicted value (BLUP) and broad-sense heritability (*H*^*2*^) of GlutoPeak parameters [41, 42]. The correlation analysis of BLUP values of each parameter was performed by IBM SPSS Statistics 22 [43].

### Genotyping and quality control

For the 207 wheat genotypes in the association population, genotyping was conducted using the wheat 660 K Illumina Infinium SNP array following the Axiom 2.0 Assay Manual Workflow protocol [21]. Genotypes were called utilizing the software obtained from commercial sources (Affymetrix and Illumina). Standardized quality control, imputation and statistical analyses were implemented. The reliable SNPs were screened by Plink version 1.9 software (re (http://www.cog-genomics.org/plink2/) w) with missingness < 0.5 and the minor allele frequency > 0.5. Finally, 224,706 SNPs remained for GWAS in the association population.

### Genome-wide association study in the association population

The Q (population structure) + K (relationship) matrix and MLM model of TASSEL 5.0 software were used for association analysis. The SNPs with -log_10_ *P* ≥ 4.0, simultaneously, the average value of each parameters detected significant difference (*t*—test, *P* < 0.05) between genotypes of the SNPs, were identified as significant SNPs [44]. The quantile–quantile plot and the Manhattan plot were created using the package “qqman”. The adjacent significant SNPs were integrated into one QTL with the physical distance of < 10 Mb, and Peak SNPs represented the SNPs with the highest phenotypic contribution. The R software package “ggplot2” was used to statistically analyze and visualize the significant SNPs. Each allele was assigned with scores: the superior alleles were given the score of 2, the inferior alleles were 0, and the heterozygous alleles were scored with 1, the scatter plot was drawn using Origin 2017.

### Genotyping of two KASP markers

Genotyping of two KASP markers in the association panel was performed using Bio-rad CFX Maestro 1.1 (Bio-Rad, California, USA) based on competitive specific amplification of allelic differential loci and fluorescence resonance energy conversion. The PCR reaction system consisted of 2.5 μl KASP Master Mix, 0.04 μl Mgcl_2_, 1ul template DNA (100 ng/μl), 0.76 μl ddH_2_O, 0.7 μl primer mixture (with 100ul mixture as the unit, the proportion of primer addition was F1:F2:*R* = 12:12:30, and the remaining volume supplemented with ddH_2_O). The PCR cycles were carried out as the following protocol: pre-denaturation at 95℃ for 15 min, denaturation at 95℃ for 20 s, annealing at 64℃ for 60 s, denaturation and annealing for 10 cycles, each cycle reduced by 1℃, then denaturation at 95℃ for 20 s, annealing at 57℃ for 60 s, denaturation and annealing for 35 cycles, the signal was read after 1 min at 37℃ [45].

### Prediction of the Candidate Genes

The genes underneath the QTL, *q3AL.2* and *q4DL*, were annotated to wheat genome reference sequence (IWGSC RefSeq v2.0), and the gene expression level in developed seeds expression were obtained through the public database of Wheat Expression Browser (http://www.wheat-expression.com) and RNA-seq data of genotypes in the association population (https://bigd.big.ac.cn/gsa/browse/CRA004223, PRJCA005188/). Spatiotemporal expression profile of the genes were analyzed. KASP markers developed from corresponding QTL and the differential expression levels of the genes in the association population, candidate genes related to GlutoPeak parameters were predicted.

**Table 1:** Variance analysis of GlutoPeak parameters in different environments

Parameters	ANOVA^a^				*H*^2 b^
	$\mathrm\sigma_{\mathrm G}^2$	$\mathrm\sigma_{\mathrm E}^2$	$\mathrm\sigma_{\mathrm{GE}}^2$	$\mathrm\sigma_{\mathrm e}^2$	
*PMT*	460.38^***^	15.24^***^	25.37^***^	3.38	0.95
*BEM*	256.66^***^	4211.69^***^	19.98^***^	3.05	0.93
*AM*	143.71^***^	886.09^***^	13.40^***^	5.5	0.91
*PM*	94.10^***^	2503.08^***^	6.22^***^	1.2	0.94
A1	378,522.10^***^	709,829.13^***^	21,409.59^***^	7398.02	0.91
A2	388,646.89^***^	378,348.94^***^	33,217.42^***^	14,742.71	0.85
A3	198,324.03^***^	58,650.03^***^	10,093.29^***^	1389.63	0.95
A4	50,392.53^***^	575,023.37^***^	3397.18^***^	1270.54	0.93
A5	28,969.37^***^	646,453.16^***^	1997.45^***^	338.59	0.94

^a^Analysis of variance of individual traits. Variance contributed by the genotypes ($\mathrm\sigma_{\mathrm G}^2$), environments ($\mathrm\sigma_{\mathrm E}^2$), genotype × environment interactions ($\mathrm\sigma_{\mathrm{GE}}^2$), and errors ($\mathrm\sigma_{\mathrm e}^2$). ^***^Variances contributed by the genotypes, environments, and genotype × environment interactions were significant (*P* < 0.001)

^b^Broad-sense heritability (*H*^*2*^) of each trait in the association population in three locations

## Supplementary Information

**Additional file1:** **Table S1. **Phenotypic values, mean values, and BLUP values of GlutoPeak parameters in the association panel under three environments. **Table S2. **Phenotypic analysis of GlutoPeak parameters in different environments. **Table S3. **Correlations between different GlutoPeak parameters. **Table S4.** SNPs significantly associated with GlutoPeak parameters. **Table S5.** QTLs for GlutoPeak parameters detected in association panel based on three environments and BLUP values. **Table S6.** Allele statistics of QTL cluster for GlutoPeak parameter. **Table S7.** KASP primers designed based on  Peak SNP sequence. **Table S8.** Analysis of variation indifferent alleles. **Table S9.** Candidate genes with significantly different expression in AX-111083649 and AX-95660756. **Table S10.** The same or adjacent QTLs in this study and previous studies.

## Data Availability

All data generated or analyzed during this study are included in this published article [and its supplementary information files] and the raw SLAF sequencing data can be found in Genome Sequence Archive (https://bigd.big.ac.cn/gsa/browse/CRA003543, PRJCA003913). The RNA-seq data are available in Genome Sequence Archive (https://bigd.big.ac.cn/gsa/browse/CRA004223, PRJCA005188/).
